# Purinergic targeting enhances immunotherapy of CD73^+^ solid tumors with piggyBac-engineered chimeric antigen receptor natural killer cells

**DOI:** 10.1186/s40425-018-0441-8

**Published:** 2018-12-04

**Authors:** Jiao Wang, Kyle B. Lupo, Andrea M. Chambers, Sandro Matosevic

**Affiliations:** 1 Department of Industrial and Physical Pharmacy, Purdue University, 575 Stadium Mall Drive, Robert E. Heine Pharmacy Building, West Lafayette, IN USA; 2 Center for Cancer Research, Purdue University, West Lafayette, IN 47907 USA

**Keywords:** NK cells, Adenosine, CD73, Chimeric antigen receptor, Immunometabolism, Cancer immunotherapy

## Abstract

**Background:**

The anti-tumor immunity of natural killer (NK) cells can be paralyzed by the CD73-induced generation of immunosuppressive adenosine from precursor ATP within the hypoxic microenvironment of solid tumors. In an effort to redirect purinergic immunosuppression of NK cell anti-tumor function, we showed, for the first time, that immunometabolic combination treatment with NKG2D-engineered CAR-NK cells alongside blockade of CD73 ectonucleotidase activity can result in significant anti-tumor responses in vivo.

**Methods:**

NK cells were engineered non-virally with NKG2D.CAR-presenting vectors based on the piggyBac transposon system with DAP10 and CD3ζ co-signaling domains. The anti-tumor immunity of NKG2D.CAR.NK cells in combination with CD73 targeting was evaluated against multiple solid tumor targets in vitro and humanized mouse xenografts in immunodeficient tumor-bearing mice in vivo. Intratumoral migration was evaluated via immunohistochemical staining, while degranulation capacity and IFN-γ production of NK cells were measured in response to solid tumor targets.

**Results:**

Our results showed that CD73 blockade can mediate effective purinergic reprogramming and enhance anti-tumor cytotoxicity both in vitro and in vivo by enhancing the killing ability of CAR-engineered NK cells against CD73^+^ solid tumor targets via mechanisms that might imply alleviation from adenosinergic immunometabolic suppression. CD73 blockade improved the intratumoral homing of CD56^+^ CAR-NK cells in vivo. These engineered NK cells showed synergistic therapeutic efficacy in combination with CD73 targeting against CD73^+^ human lung cancer xenograft models. Interestingly, CD73 blockade could inhibit tumor growth in vivo independently of adaptive immune cells, innate immunity or NK cell-mediated ADCC.

**Conclusions:**

Immunotherapies targeting the adenosinergic signaling cascade, which act by neutralizing CD73 ectoenzymatic activity, had thus far not been evaluated in humanized tumor models, nor had the implication of innate immunity been investigated. Taken together, our pre-clinical efficacy data demonstrate, for the first time, the potential of targeting CD73 to modulate purinergic signaling and enhance adoptive NK cell immunotherapy via mechanisms that could implicate autocrine tumor control as well as by mediating adenosinergic signaling.

**Electronic supplementary material:**

The online version of this article (10.1186/s40425-018-0441-8) contains supplementary material, which is available to authorized users.

## Background

Extracellular adenosine (ADO) generated in response to tumor microenvironment (TME) hypoxia has been recognized as a critical immunosuppressive metabolite that impairs anticancer immune responses [1, 2]. ADO disables cytotoxic effector functions of both CD8^+^ T and natural killer (NK) cells predominantly via A_2A_ adenosine receptor signaling, enabling tumor immune evasion and escape [3, 4]. Furthermore, signal transduction through the A_2A_ adenosine receptor inhibits the Th1 CD4^+^ T-cell response, debilitating the cytokine environment necessary to support these effector cell types [5], and enhances proliferation of regulatory T cells (T_regs_) and granulocytic MDSCs (myeloid-derived suppressor cells) [6]. Apart from its role in regulating global immune responses, ADO exerts powerful and specific immunomodulation of NK cell function [7]. Alongside inhibiting maturation of NK cells and limiting accumulation of cytotoxic CD56^dim^ subsets [4], ADO suppresses trafficking of NK cells to tumor sites by altering the chemokine milieu [8], and inhibits NK cell effector function against tumor targets [9]. Therefore, modulating ADO levels in the TME may ameliorate antitumor immunity and inhibit tumor growth. Recently, Hatfield et al. showed that downregulation of tumor-derived extracellular adenosine by oxygen supplementation improved the cytotoxic capacity of CD8^+^ T and NK cells [10]. Arabella et al. reported that co-inhibition of CD73 and adenosine A_2A_ receptor signaling enhanced anti-tumor immune responses by improving recruitment of NK cells to tumor sites [11]. It is becoming evident that targeting the adenosinergic pathway holds significant potential for cancer treatment and can enhance adoptive immunotherapy.

NK cells, specialized effectors of the innate immune system, can respond rapidly to cancer cells due to expression of germline-encoded activating receptors capable of directly binding to pathogen-derived or stress-induced self-antigens [12]. Allogeneic NK cells (such as clinically-studied NK-92 cells [13]) cause no graft versus host disease (GVHD) making their widespread, off-the-shelf use feasible [14]. Mature NK cells have a relatively limited life-span, permitting effective antitumor activity while reducing the probability of long-term adverse events, such as on-target/off-tumor effects [15]. Expression of chimeric antigen receptors (CARs) can increase the specificity and the cytotoxicity of NK cells against cancer targets [16] and rescue the downregulation of activating receptors induced by suppressive TME mechanisms such as hypoxia [17]. NK cells also have a better safety profile as they can avoid in vivo cytokine storm [18] and lack clonal expansion.

**Scheme 1:**
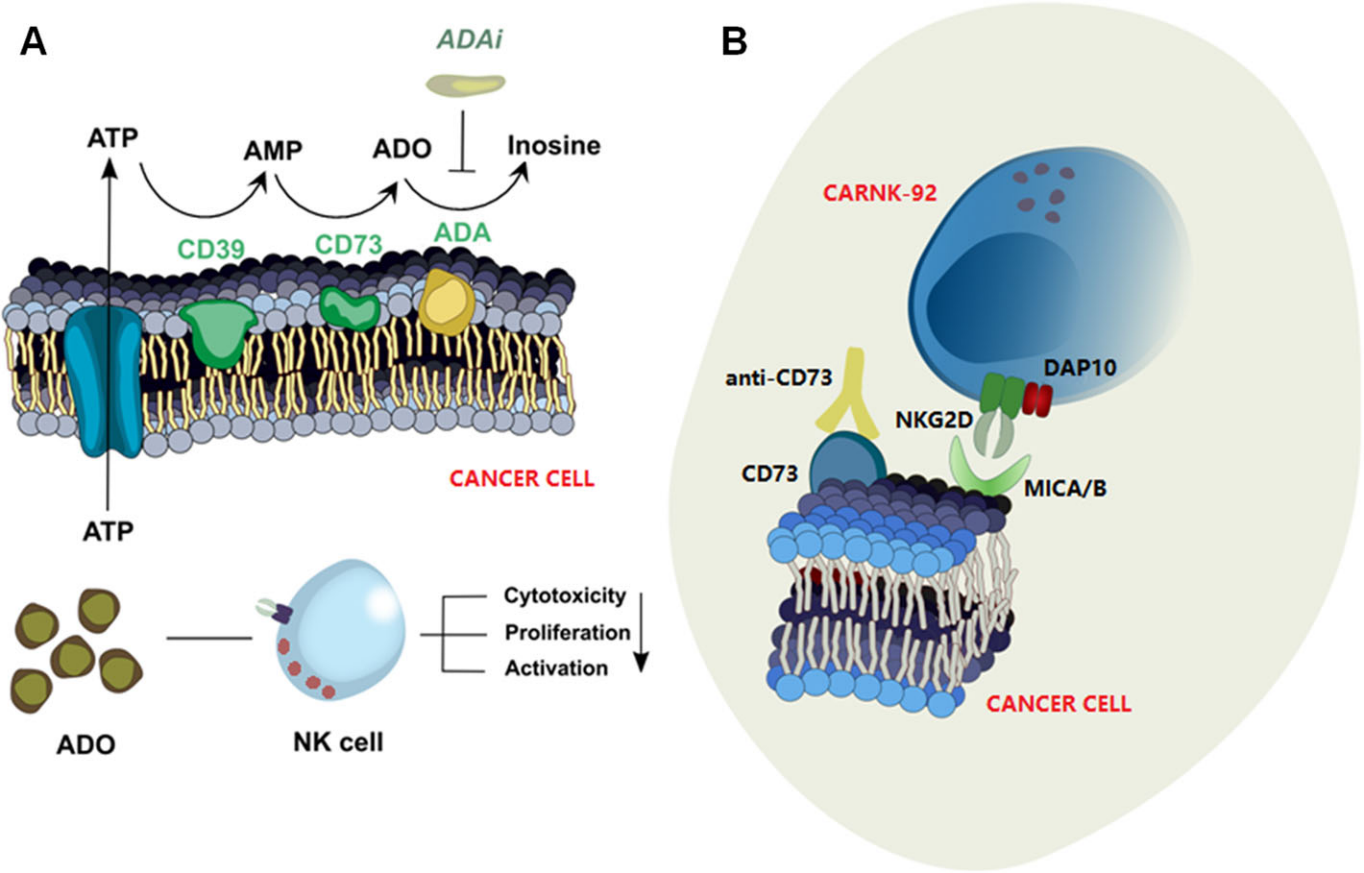
CD73-mediated adenosinergic cascade and CAR-NK immunotherapy. **a** Extracellular adenosine (ADO) metabolism in the tumor microenvironment and its inhibition of NK cell effector functions. Two ectoenzymes, E-NTPDase1 (CD39) and ecto-5’-Nucleotidase (CD73), which are highly overexpressed on many solid tumors, convert extracellular ATP to ADP/AMP and AMP to ADO, respectively, leading to elevated levels of ADO in the tumor microenvironment. ADO signals on adenosine receptors on NK cells, ultimately suppressing their immune response. The release of ADO is modulated by adenosine deaminase (ADA), which deaminates ADO to inosine. **b** Combination immunotherapy with NKG2D.CAR-NK cells and anti-CD73 antibody enhances suppression of tumor growth. NKG2D.CAR-NK cells are engineered with a synthetic immunoreceptor which recognizes MHC class I-like ligand MICA, resulting in an increase in their antitumor capacity through antigen-receptor binding, by counteracting TME-induced downregulation of NKG2D. Co-administration of anti-CD73 antibody can modulate tumor metabolism by redirecting purinergic signaling through blockade of ADO production

In the context of the relationship between adenosine immunosuppression and NK cells’ antitumor immunity, we hypothesize that purinergic signaling blockade may be able to boost cancer immunotherapy with CAR-NK cells. As shown in Scheme 1a, ADO is generated from released extracellular ATP in a stepwise manner by the ectoenzymes CD39 and CD73, respectively, leading to elevated levels of ADO in the TME. CD73 is significantly upregulated in cancerous tissues in response to hypoxia, accompanied by high enzymatic activity [19]. As regulator of this adenosinergic signaling cascade, hypoxia has the ability to downregulate activating receptors on NK cells. Moreover, CD73 expression has been shown to negatively correlate with patient prognosis in a number of cancer types [20]. Accordingly, CD73 has emerged as an important target for cancer immunotherapy owing to its role in a number of immunosuppressive mechanisms. Monoclonal antibodies targeting CD73 have shown antitumor effects [10, 21, 22]. In addition, CD73 blockade synergizes with other antineoplastic agents, such as anthracycline [23], anti-CTLA-4 [24], and anti-PD-1 [24] therapy. However, all of the effects shown in current studies have been evaluated in murine tumor models with largely intact immune systems, using mice as the species reactivity of the anti-CD73 clones. Parsing out the effect of purinergic CD73 blockade on NK cell immunotherapy has not yet been demonstrated, and though it was suggested that NK cells respond to CD73 targeting via mechanisms that are antibody-dependent cellular cytotoxicity (ADCC)-independent, no in vivo studies exist. Using CAR-NK cells in such a setting, however, is often difficult because NK cells are well known as being hard-to-transfect compared to T cells [25]. Currently, the vast majority of systems used in establishing CAR-NK cells are based on viral vectors which have serious safety implications, or non-integrating naked plasmid DNA or RNA, which induce transient gene expression [18]. It is essential to develop safe, clinically-viable and stable non-viral vector systems that generate effective CAR-NK cells.

Here, we report pre-clinical data showing that combination immunotherapy with CD73 blockade and CAR-NK-92 cells shows improved anti-cancer effects against CD73^+^ solid tumor targets both in vitro and in vivo. NK cells, engineered using a genomically-stable and clinically-safe non-viral vector system to target NKG2D ligands via DAP10 and CD3ζ co-signaling domains based on the piggyBac transposon system, could mediate a powerful anti-tumor response in conjunction with purinergic blockade (Scheme 1b). Our results revealed that CD73 targeting enhances NK cell effector function via mechanisms that are ADCC- and adaptive immunity-independent. Taken together, this is the first report demonstrating the performance of non-viral piggyBac transposon system-engineered CAR-NK cells. We also demonstrate, for the first time, that targeting the CD73-adenosine axis could be used in combination with CAR-NK cell immunotherapy to redirect purinergic signaling away from adenosinergic immunosuppression as an immunometabolic treatment of solid tumors.

## Results

### Generation of NKG2D-specific piggyBac.CAR-NK-92 cells

**Fig. 1:**
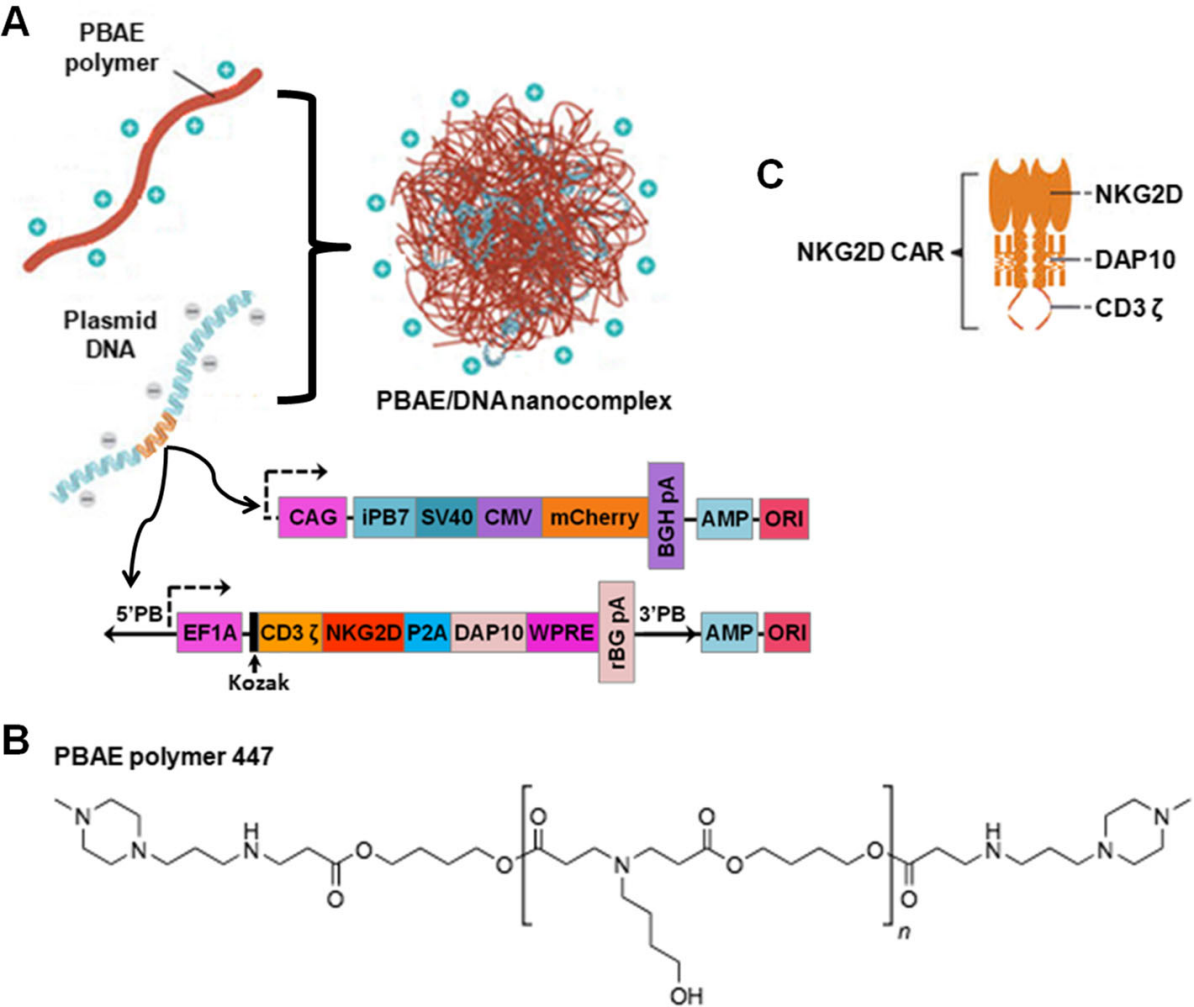
Design of NK cell-reprogramming piggyBac vector, CAR construct, and non-viral delivery platform. **a** Diagram describing the fabrication of the PBAE/DNA vector complex and structure of piggyBac CAR construct. Herein, two plasmids comprising the piggyBac transposon/transposase system encoding an NKG2D.CAR and the hyperactive iPB7 transposase, respectively, were complexed with the PBAE 447 polymer. The transposon/iPB7 transposase comprises the following genetic elements: CAG, CMV early enhancer fused to modified chicken β-actin promoter; SV40 pA, Simian virus 40 late polyadenylation signal; CMV, human cytomegalovirus immediate early enhancer/promoter; mCherry, variant of mRFP1 generated by mutagenesis; EF1A, eukaryotic translation elongation factor 1 alpha 1; Kozak, kozak consensus sequence; BGH PA, bovine growth hormone polyadenylation signal; rBG pA, rabbit β-globin polyadenylation signal; AMP, ampicillin resistance gene; ORI, origin of replication. **b** Chemical structure of the PBAE 447 polymer. **c** Schematic of the NKG2D.CAR construct containing NKG2D as the ectodomain linked to DAP10 and CD3ζ as key signaling molecules

As illustrated in Fig. 1a, we designed a clinically-safe non-viral CAR-presenting system which can mediate stable incorporation of the CAR transgene based on a PBAE 447 polymer carrier and CAR-encoding piggyBac vectors. Here, the biodegradable PBAE 447 polymer (chemical structure shown in Fig. 1b) synthesized according to previously published protocols (Additional file 1: Figure S1) [26] was used as the CAR gene carrier. The vector, referred to as the transposon plasmid, was constructed with a gene expression cassette encoding the NKG2D-specific CAR, which is a fusion receptor composed of two identical extracellular human NKG2D domains combined with DAP10 and CD3ζ signaling domains (Fig. 1c). The second vector of the dual piggyBac system, also referred to as the helper plasmid, encodes a hyperactive form of the piggyBac transposase (iPB7).

**Fig. 2:**
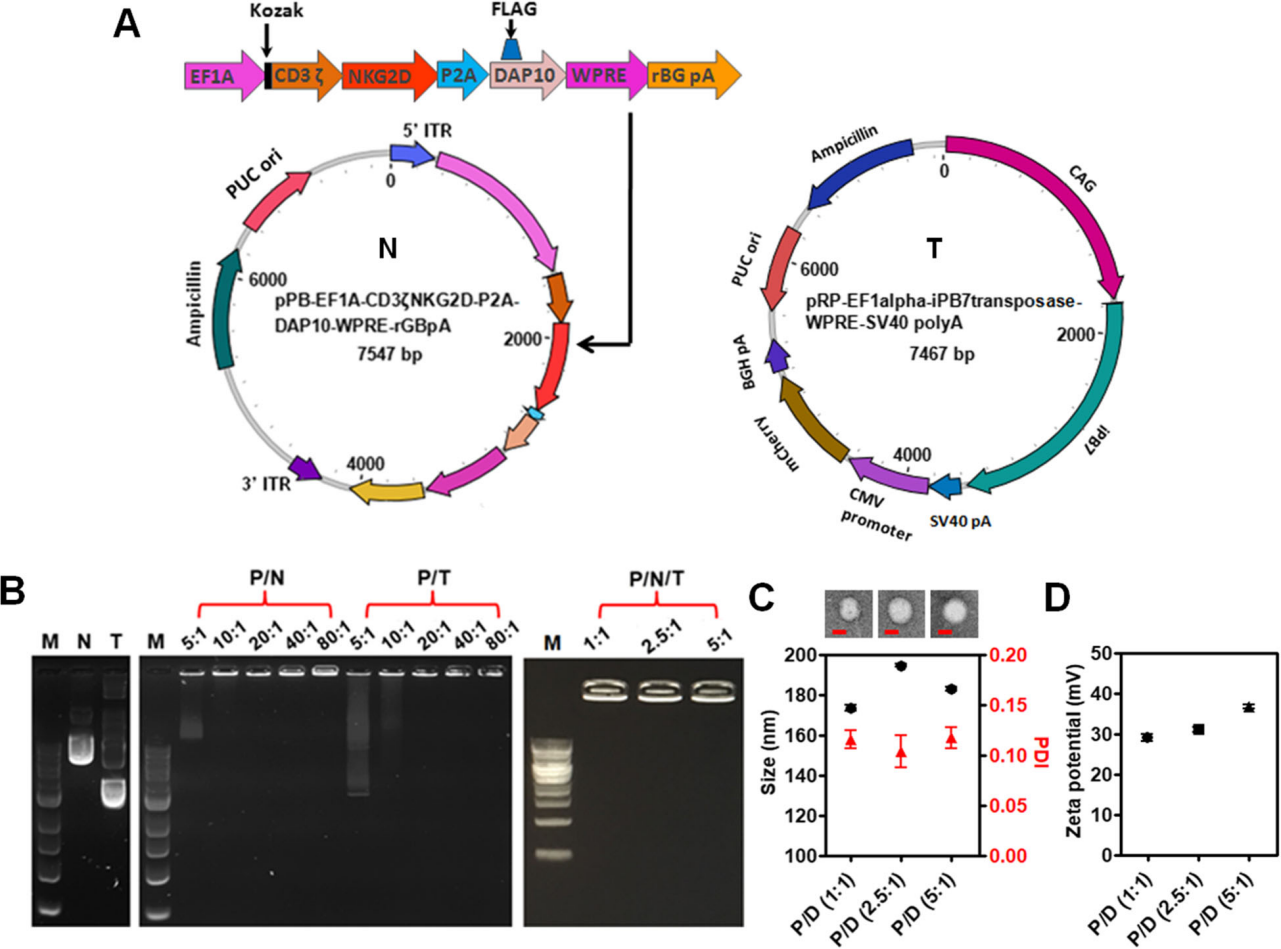
Characterization of piggyBac-NKG2D.CAR/PBAE 447 vector construct. **a** Structures of piggyBac transposon/transposase plasmids encoding CAR construct. On the left (N) is the piggyBac donor plasmid encoding the NKG2D-DAP10-CD3ζ CAR gene. On the right (T) is the piggyBac helper plasmid containing the transposase gene. **b** Agarose gel electrophoresis images of naked plasmid DNA (N and T), transposon/transposase: PBAE 447 complexes (P/N and P/T) (mass ratio of PBAE 447 to N or T is 5, 10, 20, 40, and 80, respectively), and PBAE 447/piggyBac-NKG2D.CAR complexes (P/N/T) (mass ratio of PBAE 447 to total DNAs is 40, and the mass ratio of N to T is 1, 2.5, and 5). **c** TEM images and DLS mean size of P/N/T complex. Bar = 100 nm. **d** Zeta potential of P/N/T. In both (**c**) and (**d**), the mass ratio of PBAE 447 to total DNA (N and T) is 40, and the mass ratio of N to T varies from 1, 2.5, and 5. Herein, samples are referred as P/D (1:1), P/D (2.5:1), and P/D (5:1), respectively. Data are presented as the mean ± SEM (*n* = 3)

The piggyBac plasmids (Fig. 2a) can bind effectively to PBAE 447 in sodium acetate buffer (25 mM, pH 5.0) through electrostatic interactions and form a self-assembling nano-complex formulation suitable for CAR presentation. The complexation and binding ability of PBAE 447 with plasmid DNA (pDNA) was examined by agarose gel electrophoresis. As shown in Fig. 2b, no retardation was found with pDNA alone. On the other hand, retardation mediated by PBAE 447 was complete at PBAE 447: pDNA mass ratios between 20:1 and 80:1, suggesting high affinity binding of pDNA with PBAE 447 polymer. According to the in vitro cytotoxicity of PBAE 447 towards NK-92 cells (Additional file 1: Figure S2), we chose PBAE 447 at a mass ratio of 40 (w/w) delivering 1 μg of piggyBac vectors as the optimal formulation for CAR transfection. In regard to the ratio between the transposon and transposase components of the piggyBac vector system, we prepared and tested three different PBAE 447/pDNA formulations with mass ratios of transposon to transposase vectors of 1:1, 2.5:1, and 5:1, respectively. All formulations were stable and could not undergo electrophoresis (Fig. 2b). The size and morphology of the formulations were further conformed by transmission electron microscope (TEM) imaging and dynamic light scattering (DLS). As shown in Fig. 2c, all formulations were well dispersed and were spherical in shape with an average diameter of ~180 nm. The zeta potentials of all formulations were ~30 mV (Fig. 2d).

**Fig. 3:**
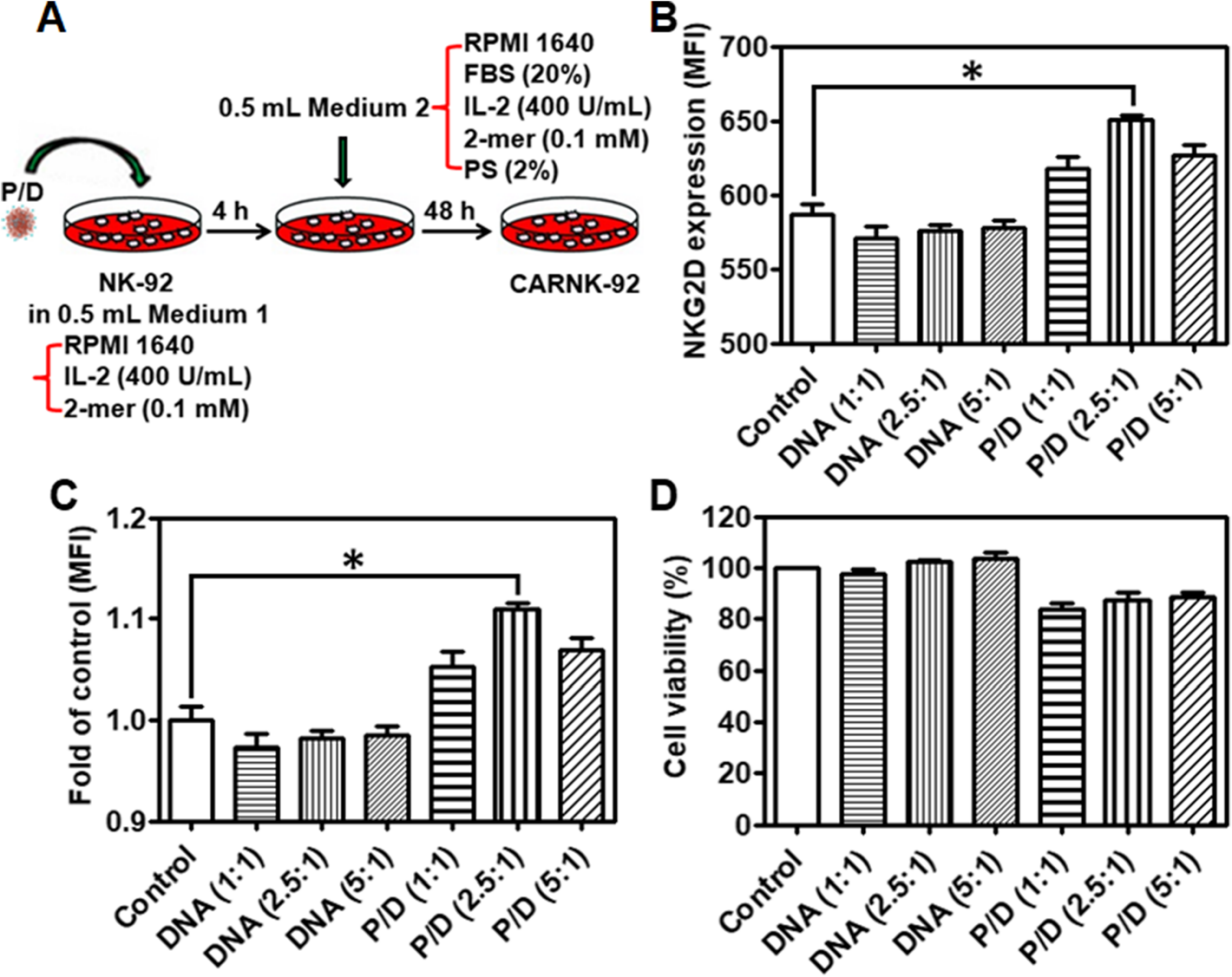
Gene transfer process and NKG2D-CAR expression. **a** Diagram of the gene transfer process with PBAE 447/piggyBac-NKG2D.CAR complex. **b** and **c** Mean fluorescence intensity (MFI) of NKG2D expression. **d** Viability of NK cells after transfection with piggyBac-NKG2D.CAR/PBAE 447 as determined by CCK-8 assay. Data are presented as the mean ± SEM (*n* = 4). *P < 0.05

NK cells were transfected with the NKG2D.CAR-presenting piggyBac plasmid via a simple gene transfer process in vitro (Fig. 3a). This method resulted in stable genomic incorporation of the CAR transgene that was dependent on the ratio of transposon and transposase plasmids. After 48 h of culture, treatment with three formulations of PBAE 447 and piggyBac vectors at 40 (w/w) with transposon and transposase plasmids at varying ratios (1:1, 2.5:1, and 5:1) resulted in the most significant increase in NKG2D expression on NK cells at a transposon:transposase ratio of 2.5:1 (Fig. 3b and c). Though NK-92 cells natively express NKG2D (Additional file 1: Figure S3), the mean fluorescence intensity (MFI) of NKG2D expression in transfected NK-92 cells was significantly above that of native NK cells (Fig. 3b and c). Biocompatibility is one of the most critical properties for the clinical viability of any gene delivery system. As shown in Fig. 3d, the cell viability of the transfected groups in the presence of all formulations of PBAE 447/CAR DNA was above 84%. Based on these results, we chose NK-92 cells transfected with PBAE 447/pDNAs with a mass ratio of the piggyBac vectors at 2.5:1 for further studies. Moreover, the MFI of NKG2D expression on expanded NK-92 cells remained consistently higher than that of untreated cells up to 5 days post-transfection (Additional file 1: Figure S4), demonstrating the stable genomic integration of CAR DNA using piggyBac vectors.

### NKG2D-specific CAR-NK-92 cells mediate effective cytotoxicity against CD73^+^ targets

**Fig. 4:**
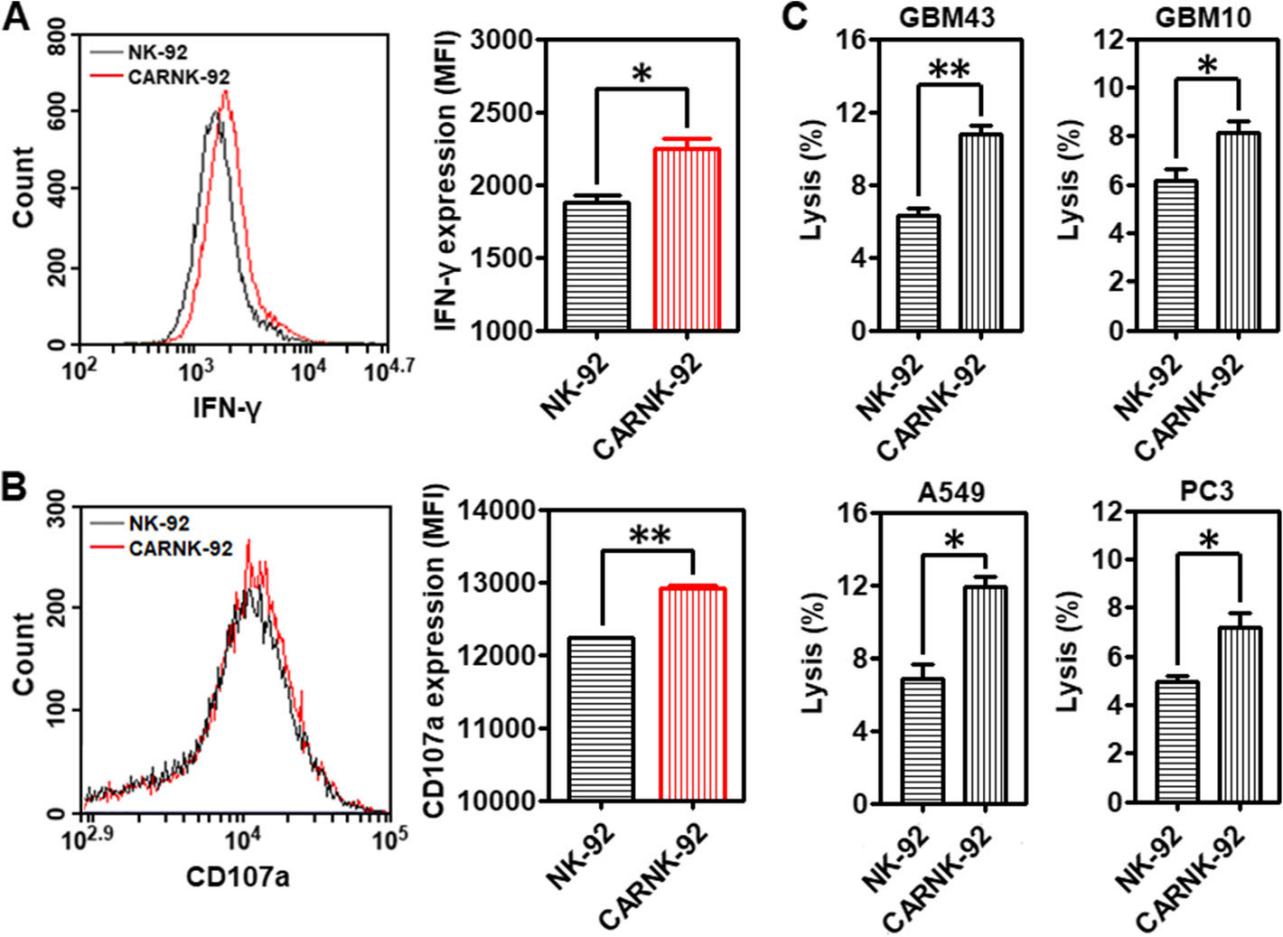
Cytotoxicity and lytic ability of piggyBac-NK2GD.CAR-NK cells against CD73^+^ targets. **a** Mean fluorescence intensity (MFI) of intracellular IFN-γ production by both NK-92 and piggyBac-NKG2D.CAR-NK-92 cells. **b** Degranulation as measured via CD107a expression (MFI) by both NK-92 and piggyBac-NKG2D.CAR-NK-92 cells. **c** Lytic activity of NK-92 and piggyBac-NKG2D.CAR-NK-92 cells against CD73^+^ GBM43, GBM10, A549 or PC3 cells, respectively. Data are presented as the mean ± SEM (*n* = 4). *P < 0.05, **P < 0.01

NKG2D is a key receptor for NK cell activation [27]. Its expression is significantly decreased by transforming growth factor β1 (TGF-β1), an important component contributing to the suppressive solid tumor microenvironment (Additional file 1: Figure S5). We found that alongside hypoxia, adenosine might contribute to the regulation of NKG2D on NK cells [28]. To investigate whether the CAR-expressing NK-92 cells, referred to as NKG2D.CAR-NK-92, could specifically recognize and be activated by CD73^+^ cancer cells, we incubated effector cells NKG2D.CAR-NK-92 or NK-92 cells alone with A549 cells at an E/T ratio of 10:1 for 4 h, following which the cells were collected for flow cytometry analysis. NKG2D.CAR-NK-92 cells respond to stimulation by CD73^+^ cancer cells by enhancing the production of cytokines. As shown in Fig. 4a and Additional file 1: Figure S6A, stimulation by cancer cells contributed to an increased intracellular production of IFN-γ by NKG2D.CAR-NK-92 cells (MFI: *P < 0.05; IFN-γ^+^ (%):*P < 0.05). In addition, exocytosis of lytic granules containing granzymes and perforin is a prerequisite for the killing ability of NK cells, with CD107a molecules appearing temporarily on the surface. Their expression can be detected as a read-out system for NK cell degranulation [29]. As shown in Fig. 4b and Additional file 1: Figure S6B (**P < 0.01; *P < 0.05**)**, NKG2D.CAR-NK-92 cells displayed significantly enhanced surface CD107a expression in response to the target A549 cells).

Patient-derived recurrent (GBM10) and primary (GBM43) glioblastoma cells, lung carcinoma cells (A549), and prostate cancer cells (PC3) were maintained in culture prior to analysis. Among these, GBM43 is a primary glioblastoma originally derived from a 69-year-old man who underwent resection of a left temporal glioblastoma. It carries mutant p53 and CDKNA deletion. GBM10, on the other hand, was derived from a recurrent wild-type p53 GBM showing high temozolomide (TMZ) and ionizing radiation resistance. All of them have been maintained as a part of a human cancer xenograft panel as previously described. The killing ability of NKG2D.CAR-NK-92 and NK-92 cells against these CD73^+^ solid tumor targets in vitro was measured using a 7-AAD/CFSE cell-mediated cytotoxicity assay. Effector cells, either NKG2D.CAR-NK-92 or NK-92 cells, were separately co-incubated with GBM43, GBM10, A549 or PC3 cells at an E/T ratio of 10:1 for 4 h. NKG2D.CAR-NK-92 cells induced a significantly higher percentage of cytolysis towards all solid tumor cell lines when compared to NK-92 cells not bearing the NKG2D.CAR (Fig. 4c; *P < 0.05, **P < 0.01).

### Targeting the CD73-purinergic cascade improves in vitro cytotoxicity of NKG2D.CAR-NK-92 cells

**Fig. 5:**
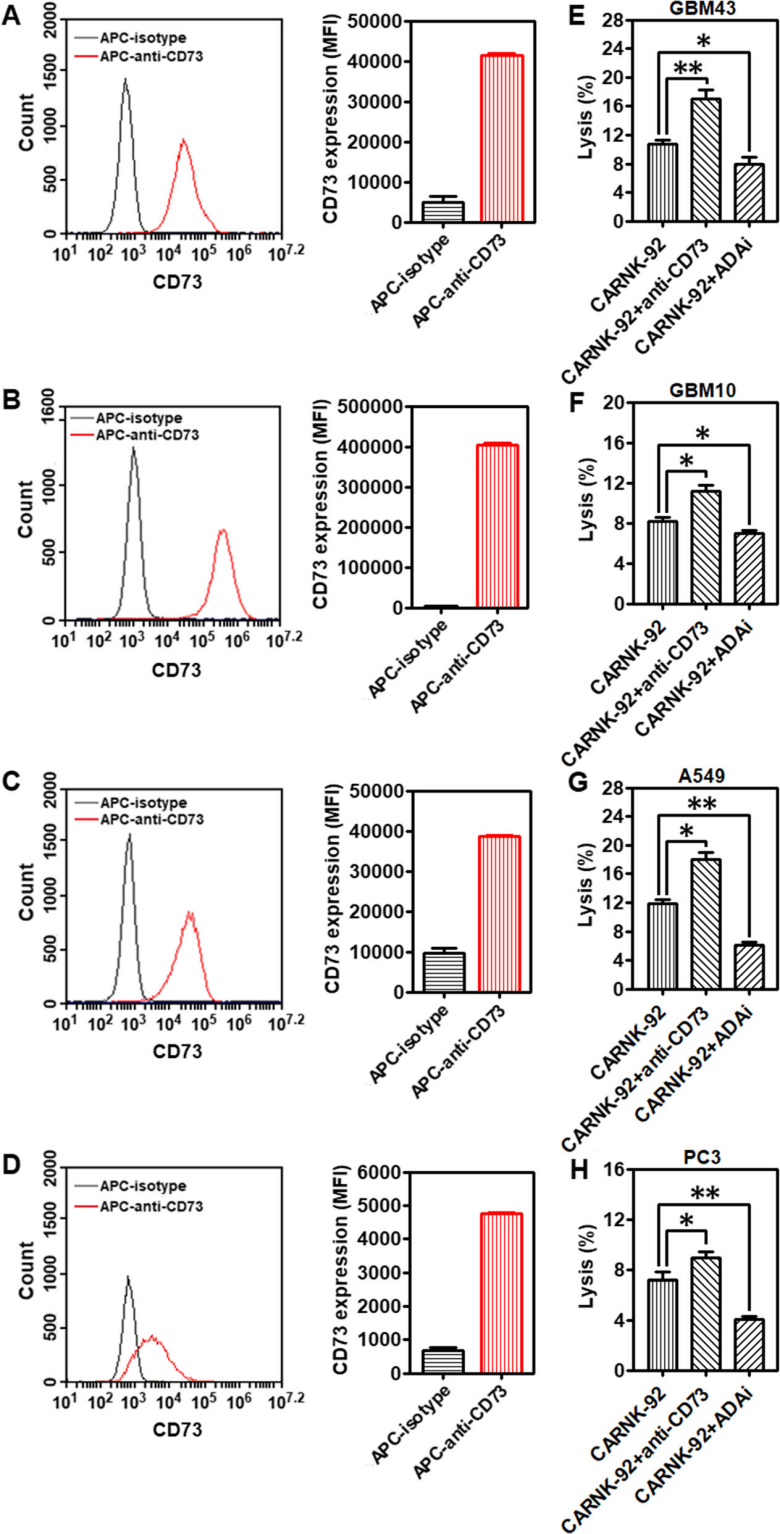
Targeting CD73 improves cytolytic activity of piggyBac-NKG2D.CAR-NK cells. CD73 expression levels on (**a**) GBM43, (**b**) GBM10, (**c**) A549, and (**d**) PC3 cell lines, respectively. Lytic activity of piggyBac-NKG2D.CAR-NK-92 cells against (**e**) GBM43, (**f**) GBM10, (**g**) A549 or (**h**) PC3 cells, in the presence of anti-CD73 antibody and adenosine deaminase inhibitor (ADAi) EHNA (30 μM), respectively. Data are presented as the mean ± SEM (*n* = 4). *P < 0.05, **P < 0.01

Cell-surface expression of CD73 was analyzed by flow cytometry on GBM43, GBM10, A549, and PC3 cells, respectively. In vitro, all the cells express high levels of CD73 (Fig. 5a-d). Catalytically, the ectonucleotidases CD73 participates in a purinergic enzymatic cascade that is responsible for the generation of extracellular ADO, which has been recognized as a potent immunosuppressor that accumulates during tumor growth [20], and is able to modulate NK cells’ anti-tumor response. High concentrations of ADO were able to cause significant inhibition of NK-92 cell proliferation (Additional file 1: Figure S7). EHNA, a specific inhibitor of adenosine deaminase (ADA), which metabolizes accumulating ADO into inosine, can contribute to a higher accumulation of ADO. As shown in Fig. 5e-h, treatment with EHNA (30 μM) decreased the killing ability of NKG2D.CAR-NK-92 cells against all the testing CD73^+^ solid tumor cells (GBM43: *P < 0.05; GBM10: *P < 0.05; A549: **P < 0.01; PC3: **P < 0.01), further confirming the impairment of anti-cancer immunity by ADO. Targeting CD73 via antibody blockade would similarly prevent accumulation of extracellular ADO and retain NK cell functionality. To investigate whether anti-CD73 antibody could increase NKG2D.CAR-NK-92 cell-mediated cytotoxicity, we co-cultured effector NKG2D.CAR-NK-92 cells in the presence of GBM43, GBM10, A549 or PC3 cells at an E/T ratio of 10:1 in presence of anti-CD73 antibody (10 μg/mL) for 4 h, respectively. NKG2D.CAR-NK-92 cells, in the presence of anti-CD73 antibody, showed a significantly higher percentage of lysis of CD73^+^ targets (GBM43: **P < 0.01; GBM10: *P < 0.05; A549: *P < 0.05; PC3: *P < 0.05) (Fig. 5e-h). In addition, our data also show both EHNA and anti-CD73 antibody treatments facilitate the killing ability of control unmodified NK-92 cells with a similar trend as that observed for engineered NKG2D.CAR-NK-92 cells (Additional file 1: Figure S8). The extent of ADA inhibition is related to adenosine metabolism by cancer cells and is different for various solid tumor targets.

### Combination with anti-CD73 targeting improves the antitumor activity of NKG2D-specific CAR-NK-92 cells in a xenograft mouse model

**Fig. 6:**
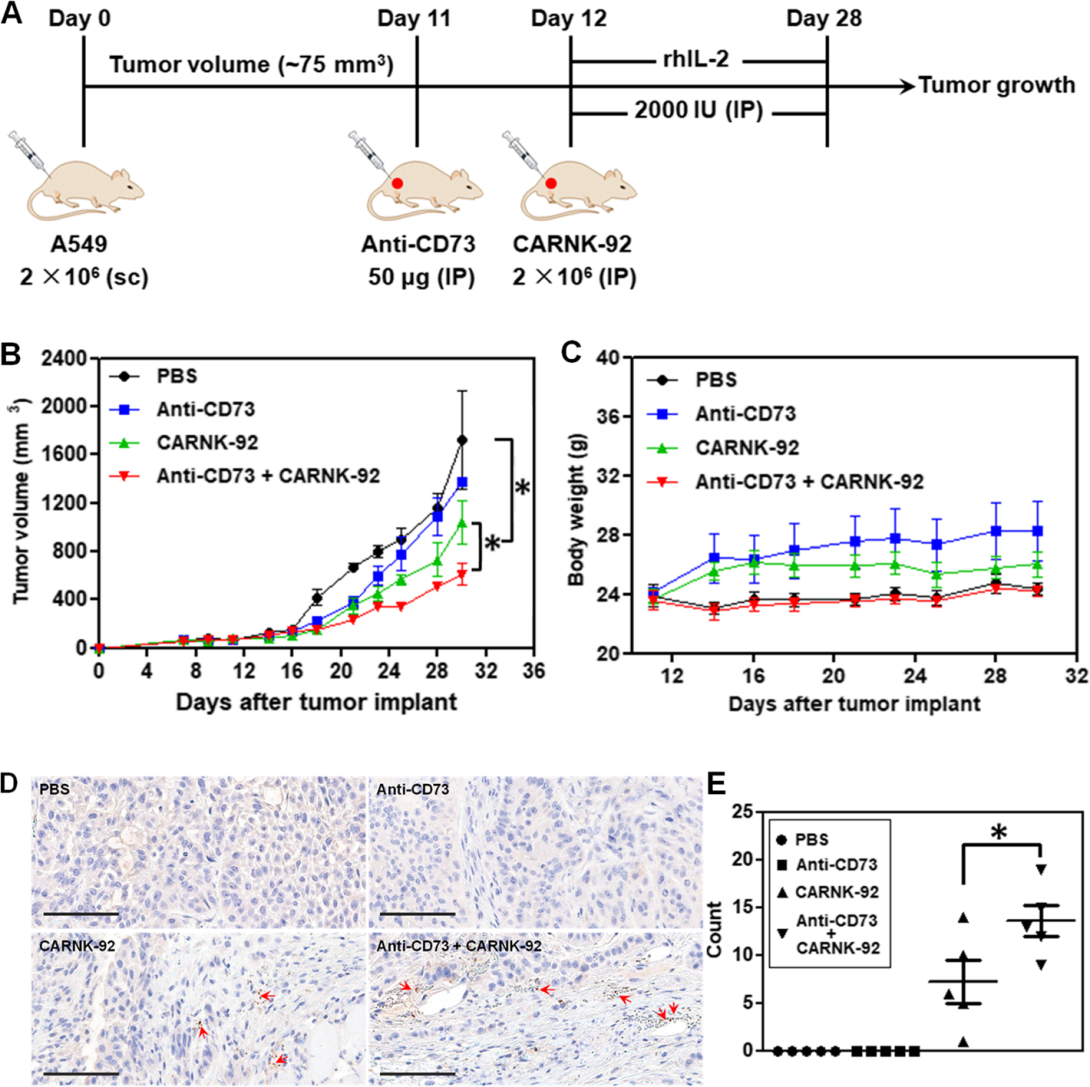
Therapeutic efficacy of piggyBac-NKG2D.CAR-NK cells combined with anti-CD73 antibody blockade for CD73^+^ tumor targeting in a xenograft mouse model. **a** Schematic diagram showing the in vivo treatment program. **b** Tumor growth curves during CAR-NK and anti-CD73 adoptive transfer immunotherapy. **c** Changes of body weights of the mice during the experiment. **d** Immunohistochemical staining for CD56^+^ NK-92 cells in tumors (arrows; magnification × 200; bar = 100 μm). **e** The corresponding quantitative analysis results of human CD56^+^ NK-92 cells. Data are presented as the mean ± SEM (*n* = 5). *P < 0.05

To further assess whether targeted blockade of CD73 could enhance the in vivo anti-tumor activity of NKG2D.CAR-NK-92 cells, we established a subcutaneous xenograft model in NSG mice with CD73^+^ human lung carcinoma A549 cells. The treatment program of the mice is shown in Fig. 6a. Briefly, 2 × 10^6^ A549 cells were subcutaneously injected (sc) into the right flank of the mice (day 0). 11 days later (day 11), the mice from both anti-CD73 antibody and anti-CD73 antibody + NKG2D.CAR-NK-92 groups started to receive anti-CD73 antibody treatment by intraperitoneal injection (IP) for 3 weeks, once a week. One day later (day 12), the mice in the NKG2D.CAR-NK-92 and anti-CD73 antibody + CAR-NK-92 groups started to receive 5 × 10^6^ NKG2D.CAR-NK-92 cell therapy (IP), respectively, once a week, 3 times in total. Starting on the day of the first injection of NKG2D.CAR-NK-92 cells, all mice were IP administered 2000 IU recombinant human IL-2 (rhIL-2) once every two days. Cytokine administration continued throughout the treatment period. All treatments ended on day 30. Treatment with anti-CD73 antibody alone significantly delayed tumor growth during the first 7-10 days of treatment, but retarded its therapeutic effect once tumor burden increased (Fig. 6b). On the other hand, NKG2D.CAR-NK-92 cells mediated potent anti-tumor responses and significantly reduced the tumor growth rate compared to the PBS group (mean tumor volume: 1039 mm^3^ vs 1721.5 mm^3^ respectively, *P < 0.05). NK cells alone were also superior in delaying tumor growth compared to anti-CD73 treatment. In addition, synergistic effects obtained by combining anti-CD73 antibody with NKG2D.CAR-NK-92 cells showed a remarkable and significantly higher delay in tumor growth compared to NKG2D.CAR-NK-92 cells alone (mean tumor volume: 611.5 mm^3^ vs 1039 mm^3^, *P < 0.05) (Fig. 6b**)**. Meanwhile, there was no significant decrease in body weight of the mice in all four groups throughout the entire treatment period (Fig. 6c). Infiltration of NK-92 cells into tumors was investigated by immunohistochemical (IHC) staining. As shown in Fig. 6c, no stained NK cells were observed in tumor samples from the PBS and anti-CD73 antibody-treated mice. However, some NK cells were observed in the tumor tissues of both the NKG2D.CAR-NK-92 group and particularly anti-CD73 antibody + NKG2D.CAR-NK-92 cell-treated group. Statistical results showed that the number of NK cells in the tumors of the anti-CD73 antibody + NKG2D.CAR-NK-92 cell-treated group was significantly higher than that in the tumors of the NKG2D.CAR-NK-92 cell-treated group, suggesting anti-CD73 antibody treatment can enhance the infiltration of NK-92 cell in CD73^+^ human lung solid tumors.

Additionally, we found that TGF-β1 can induce an increase in CD73 expression on both A549 and GBM10 solid tumor cells, which was particularly significant in the case of A549 cells (Additional file 1: Figure S9). This suggests that the TME could drive solid tumor tissues to be more CD73 positive, thus enabling higher levels of adenosine production, ultimately enhancing immunosuppression. Based on the results, CD73 appears to have an increasingly critical role in solid tumor progression and is a novel target for cancer immunotherapy.

## Discussion

NK cells, innate lymphocytes, play a primordial role in tumor immunosurveillance and can detect and eliminate cancer cells that are deficient in MHC class I molecules [30]. Given their potent anticancer activity, therapeutic manipulation of NK cells provides an attractive strategy for cancer treatment. NKG2D, the best characterized among NK cells’ activating receptors, is directly involved in the recognition of tumor cells that express its ligands [16, 27, 30]. However, immunosuppressive cytokines and metabolites in the TME, such as TGF-β and ADO, alongside hypoxia, inhibit NK cell function by downregulating NKG2D alongside other activating receptors, leading tumors to escape surveillance by NK cell-mediated responses [31–34]. To mitigate loss of NKG2D, NK cells have been engineered with CARs that express NKG2D ligands fused to various co-signaling domains [35]. Endowing NK cells with endogenous transgenes encoding NKG2D enables them to restore the loss in NKG2D expression induced by the tumor microenvironment. It also enables them to maintain, or increase, NKG2D expression to promote sustained cytolytic ability. In order to enhance the clinical feasibility and improve the safety of adoptively-transferred, engineered NK cells, we developed non-viral, allogeneic NKG2D-CAR NK cells using PBAE447 and CAR-encoding piggyBac vectors, which bear the ability to induce increased and stable NKG2D expression in NK-92 cells via the genomic incorporation of CAR transgenes. We observed significantly more robust and sustained expression of NKG2D over time in transfected cells compared to non-transfected controls. In addition, preliminary data with primary human-derived NK cells showed an increase in NKG2D expression in transfected cells using the PBAE447/CAR-encoding piggyBac-based system (data not shown). This suggests that piggyBac-based genetic modification of NK cells could also be expanded to the autologous use of patient-derived cells. NKG2D.CAR-NK-92 cells also consistently showed higher degranulation activity, IFN-γ production and killing ability against CD73^+^ solid tumor targets (GBM43, GBM10, A549, and PC3). Besides, these NKG2D.CAR-NK-92 cells also showed stronger lytic activity against CD73^-^ liquid tumor cells (K562) (Additional file 1: Figure S10**)**.

However, TME immunosuppression operates across a broad spectrum of mechanisms which collectively inhibit NK cell anti-tumor responses. Among these, the TGF-β pathway has been extensively characterized [36], while ADO, a purine metabolite, has more recently emerged as a potent immunosuppressant. ADO, which is generated and accumulates in the hypoxic TME, suppresses NK cells’ migration into tumor sites, while elevated concentrations of ADO can significantly suppress the proliferation of NK-92 cells. Activation with cytokines such as IL-2 is likely to be dampening ADO-induced immunosuppression, as evidenced by the high concentrations of ADO needed to achieve sustained inhibition of NK cell proliferation.

One of ADO’s key signaling mechanisms is via interaction with adenosine receptors, a group of four G-protein-coupled receptors, most notably the A_2A_ adenosine receptor, causing dysregulation of effector immune cell subsets (including tumor-infiltrating NK cells and CD8^+^ T cells), dampening their antitumor immune response [37]. This was evident by the effect of EHNA treatment in our study, which induced a significant decrease in the lytic activity of IL-2-primed NKG2D.CAR-NK-92 cells. EHNA can rescue ADO immunosuppression by inhibiting the enzyme adenosine deaminase (ADA), which converts ADO into inosine, leading to the extracellular accumulation of higher concentrations of ADO. In addition, ADO can directly regulate tumor proliferation, survival, adhesion, and migration by binding to A_2B_ adenosine receptors, which are expressed on cancer cells [38]. Based on the functions of ADO through the activation of distinct adenosine receptors, therapeutic strategies targeting adenosine receptors that inhibit ADO signal transduction are showing great potential. Among these, A_2A_ antagonists have been shown to be able to enhance anti-tumor immunity [39]. Moreover, some of them are undergoing clinical testing (NCT02655822, NCT02403193, and NCT02740985).

ADO accumulates upstream of its signaling interactions via its receptors, through an enzymatic cascade that concludes with CD73, an ectonucleotidase which converts AMP into ADO. This cascade makes CD73 a key modulator of the high levels of ADO in the TME of solid tumors. Overexpression of CD73 has been found in broad types of cancer cell lines and patients’ biopsies including breast cancer, colorectal cancer, ovarian cancer, gastric cancer, glioblastoma and gallbladder cancer [20]. Moreover, its expression is associated with poor clinical prognosis of many cancer patients [40]. In our study, all of the tested cancers cells, including patient-derived glioblastoma (GBM43 and GBM10), lung carcinoma (A549), and prostate cancer (PC3) cells were characterized by high CD73 expression. In addition to the immunoregulatory roles of CD73, the activity of CD73 was also revealed to be related to cancer proliferation, differentiation, invasion, and metastasis. CD73 induces the release of matrix metalloproteinases (MMPs) that facilitate breakdown of the extracellular matrix (ECM), thus enabling cancer cell invasion and migration [41, 42]. CD73 promotes cervical cancer cell proliferation and migration, via potentiating EGFR/Akt and VEGF/Akt pathways [43]. Moreover, CD73 regulates stemness and epithelial-mesenchymal transition (EMT) in ovarian cancer-initiating cells [44]. High CD73 expression is mainly driven by TME hypoxia, through a hypoxia-inducible factor 1α (HIF-1α) binding site in the CD73 gene promoter [45]. Hypoxia causes impairment of NK cell functions and downregulation of activating receptor NKG2D [17, 46]. Compounding this is TGF-β, which can increase CD73 expression on tumor-infiltrating cells [47]. Our data suggests that CD73 expression on CD73^+^ solid tumor cells (both GBM10 and A549) can be further increased due to TGF-β. Collectively, this creates a solid tumor niche that fuels cancer progression via mechanisms that lead to higher ADO concentrations and impaired NK cell functions. In this context, CD73 has risen to prominence as a biomarker and a meaningful target for cancer therapy.

In response to this, antibody-mediated CD73 blockade has been reported to show anti-cancer effects when used individually or in combination with other agents [11, 21, 22, 48]. So far, one human CD73 antibody (MEDI9447) has progressed to clinical trials (NCT02503774). However, most of the studies have been limited to evaluating the efficacy of CD73 blockade in murine tumor models, using mouse as the species reactivity of the anti-CD73 clones. Previously, Sebastian *et al*. showed that anti-human CD73 antibody could improve NK cell killing against CD73^+^ ovarian cancer cells in vitro by acting on mechanisms that may be ascribed to both ADCC and reduced adenosine levels [49]. In our study, we used an anti-human CD73 antibody (clone 7G2) which can bind specifically to CD73 and neutralize the biological activity of CD73. We found that treatment with anti-CD73 antibody could inhibit A549 tumor growth in vivo independently of adaptive immune cells or NK cells in the early stages of tumor growth. At the doses used in our study in vivo, once tumor burden increased, the effect of CD73 lessened. These results could indicate that while CD73 has the ability to act in an autocrine manner to promote tumor progression—thus supporting recent findings on the mechanistic action of CD73 on solid tumors [43, 44]—treatment regimen, dosage, and antibody isotype might play a role in controlling its therapeutic effect. Nonetheless, treatment with NKG2D.CAR-NK-92 cells in combination with CD73 blockade induced a sustained, consistent, and significantly heightened suppression of tumor growth. Because NK-92 cells used in this study do not express the FcγRIIIa receptor (CD16), they are unable to mediate ADCC [50], suggesting that mechanisms other than recruitment of NK cell activity via ADCC engagement enables altered anti-tumor function in response to blockade of CD73. This was supported by an enhanced intratumoral migration of engineered NK cells into CD73^+^ lung tumors in combination with CD73 blockade. Purinergic targeting via CD73 might thus be regulating tumor growth also via mechanisms that exclude immune cell recruitment, a finding that has important clinical implications.

## Conclusions

Our results show that targeting CD73 activity can redirect purinergic metabolism and enhance immunotherapy of CD73^+^ solid tumors—including patient-derived primary and recurrent targets—with engineered NK cells by alleviating TME immunometabolic suppression induced by adenosinergic signaling, and enhance intratumoral migration of NK cells in vivo. We observed that hypoxia-induced CD73 ectonucleotidase activity is able to mediate both tumor control as well as innate immunity. By engineering NK cells non-virally using piggyBac vectors, we demonstrated the pre-clinical feasibility of a new cell engineering approach that can mediate stable genomic incorporation of CAR transgenes as a directly translatable immunotherapy of solid tumors.

## Materials and Methods

### Mice

Male 6- to 8-week-old NOD.Cg-Prkdc^scid^ IL2rg^tm1Wjl^/SzJ (NSG) mice were maintained at the Purdue Center for Cancer Research. All the animal experiments described in this study were approved by the Purdue University Animal Care and Use Committee.

### Cell culture

NK-92 cells (directly purchased from ATCC) were maintained in RPMI 1640 supplemented with 10% FBS, 100 U/mL penicillin, 100 μg/mL streptomycin, 2 mM L-glutamine, 400 U/mL rhIL-2, and 0.1 mM 2-mercaptoethanol. GBM43 and GBM10 cells (kindly provided by Dr. Karen E. Pollok, Indiana University School of Medicine) were grown in DMEM supplemented with 10% FBS and 1% HEPES. All cell lines were incubated at 37 °C in a humidified 5% CO_2_ environment. A549 cells (kindly provided by Dr. Darci Trader, Purdue University) were grown in DMEM supplemented with 10% FBS, 100 U/mL penicillin, and 100 μg/mL streptomycin. PC3 cells (kindly provided by Dr. Marxa L. Figueiredo, Purdue University) were grown in RPMI1640 supplemented with 10% FBS, 100 U/mL penicillin, and 100 μg/mL streptomycin.

### Materials

1,4-butanediol diacrylate (90%), 4-amino-1-butanol (98%), diethyl ether (≥ 99.7%), PBS (10×), and EHNA hydrochloride (≥ 97%) were purchased from Millipore Sigma (USA). 1-(3-aminopropyl)-4-methylpiperazine (98%) was purchased from Alfa Aesar (USA). Dimethyl sulfoxide-D6 (DMSO-D6) (99.9%) was purchased from Cambridge Isotope Laboratories (USA). Tetrahydrofuran (THF), dimethyl sulfoxide (DMSO), and tris-borate-EDTA (TBE) (10×) were purchased from ThermoFisher Scientific (USA). Recombinant human IL-2 (rhIL-2) was gifted from Akron Biotech (USA). RPMI 1640, DMEM, penicillin/streptomycin solution 100× (PS), 2-mercaptoethanol (2-mer, 50 mM), HEPES (1 M), and trypsin-EDTA were from Gibco (ThermoFisher Scientific, USA). Fetal bovine serum (FBS) was purchased from Corning (USA).

### Generation of NKG2D CAR construct

All of the plasmids and CAR constructs used in this project were custom-cloned by vectorbuilder.com. The following piggyBac transposon gene expression vectors were used:

1. pPB-EF1A > hCD247:hKLRC4-KLRK1:P2A:FLAG/hHCST:WPRE
  In this construct, a human NKG2D-specific CAR (NKG2D-DAP10-CD3ζ) was expressed under the control of the EF1alpha promoter. The NKG2D sequence was derived from previous work.
2. pRP-mCherry-CAG > hyPBase
  This plasmid encodes the hyperactive version of piggyBac transposase under the control of the CMV promoter.

### PBAE 447 synthesis

This polymer was synthesized using a two-step reaction method according to previous reports [26]. Briefly, 1,4-butanediol diacrylate was mixed with 4-amino-1-butanol in a 1.1:1 molar ratio of diacrylate monomer to amine monomer. And the mixture was heated to 90 °C with stirring for 24 h to yield acrylate-terminated base polymer, and 2.3 g of this polymer was dissolved in 2 ml THF. To form the piperazine-capped polymer, 786 mg of 1-(3-aminopropyl)-4-methylpiperazine was dissolved in 13 mL THF, and then added to the base polymer/THF solution. The resulting mixture was stirred at RT for 2 h, then the capped polymer was precipitated with 5 volumes of diethyl ether. After the solvent was decanted, the polymer was washed with 2 volumes of fresh ether, then the residue was dried under vacuum for 2 days before it was formed into a stock of 100 mg/mL in DMSO, which was stored at −20 °C.

### PBAE 447/CAR complex formation and characterization

All components were diluted in sodium acetate buffer (25 mM, pH 5.2) to the following concentrations: PBAE 447 (8, 4, 2, 1, and 0.5 μg/μL); N (0.1 μg/μL); and T (0.1 μg/μL). A series of PBAE/DNA complexes were prepared by adding the PBAE 447 solution into DNA solution. Then the mixture was vortexed gently for 10 s then incubated at RT for 15 min. The complexes were subjected to electrophoresis to examine the association between the polymer and DNA. Herein, electrophoresis was performed on 1% agarose gel and run in TBE buffer at 90 V for 1 h. To characterize the morphology of the corresponding composites, transmission electron microscope (TEM) was performed on FEI Tecnai G2 20, 200 kV. Dynamic light scattering system (DLS, Malvern Zetasizer Nano ZS, USA) was used to measure particle size and zeta potential.

### CAR gene transfer and expression

NK-92 cells were suspended in RPMI 1640 medium without FBS and PS and seeded into 12-well plates (0.5 mL/well, 4 × 10^5^ cells). PBAE/DNA complexes (total DNA amount: 1 μg/well) were then added and incubated for 4 h. After that, 0.5 mL of RPMI 1640 medium with FBS (20%), rhIL-2, 2-mer, and PS (2%) were added to change the final medium back to the normal completed medium and the NK/gene complexes were cultured for additional 48 h. To measure the levels of NKG2D transfection, the cells were stained with FITC-conjugated NKG2D antibody (clone 1D11, BioLegend) and analyzed by flow cytometry using BD LSRFortessa (Becton Dickinson). The cell viability after transfection was measured by CCK-8 (Dojindo Molecular Technologies, Inc) assay analysis.

### IFN-γ release assay

Target cells (A549) were labeled with CFSE and seeded into 12-well plates. NK-92 and CAR-transfected NK-92 cells (referred to as CAR.NK-92) were then added at a E:T ratios of 10:1. To measure IFN-γ production, GolgiPlug (BD Biosciences) was added followed with the addition of NK cells. After co-culture for 4 h, the cells were collected and fixed with Cytofix-Cytoperm (BD Biosciences) for 20 min at 4°C. Then, the cells were treated with PerCP-Cy5.5-conjugated IFN-γ antibody (clone B27, BD Biosciences) in Perm/Wash buffer (BD Biosciences) for 30 min at 4°C. After that, the cells were collected and analyzed by flow cytometry using BD Accuri C6 Plus (Becton Dickinson).

### Natural killer cell degranulation and cytotoxicity

CFSE-labeled target cells, seeded into 12-well plates as before, were incubated with NK-92 or CAR.NK-92) at an E:T ratio of 10:1.To detect NK cell degranulation via CD107a staining, PE-conjugated-CD107a antibody (clone H4A3, BioLegend) was added at the beginning of the assay. After 1 h of co-incubation, GolgiStop (BD Biosciences) was added. After incubation for an additional 3 h, the cells were collected and analyzed by flow cytometry using BD Accuri C6 Plus (Becton Dickinson). NK cell-mediated cytotoxicity against tumor cells (GBM43, GBM10, A549 or PC3) was analyzed using a 7-AAD/CFSE assay (Cayman Chemical). Briefly, target cells were labeled with CFSE and seeded into 24-well plates. Then the plates were placed in the incubator for at least 4 h to allow for cell attachment. After that, NK-92 and CAR.NK-92 were added at a E:T ratios of 10:1, and co-cultured with target cells for 4 h. Finally, the cells were collected and stained with 7-AAD and cytotoxicity was measured by flow cytometry using BD Accuri C6 Plus (Becton Dickinson).

### CD73 expression and lytic activity

Detached GBM43, GBM10, A549 or PC3 cells (10^6^/sample) were stained with APC-conjugated mouse anti-human CD73 (clone AD2, BD Biosciences) and APC mouse IgG1, κ isotype (clone MOPC-21, BioLegend) for 30 min at 4 °C, respectively. Then, cells were assessed for expression of CD73 using a BD Accuri C6 Plus cytometer (Becton Dickinson). The lytic activity of CAR-NK-92 cells against GBM43, GBM10, A549 or PC3 cells in the presence of anti-CD73 (clone 7G2, ThermoFisher Scientific) or an adenosine deaminase inhibitor (EHNA hydrochloride) was analyzed using the 7-AAD/CFSE assay according to the procedure described earlier in Materials and Methods (2.9). The final concentrations of anti-CD73 antibody and EHNA hydrochloride were 10 μg/mL and 30 μM, respectively.

### In vivo efficacy studies

6-week-old male NSG mice (*n* = 25) were subcutaneously (sc) inoculated with 2 × 10^5^ A549 cells, suspended in 100 μL of DMEM medium without FBS and PS, dorsally on the right side. When tumors reached a volume of about 75 mm^3^, mice were randomly assigned to 4 treatment groups (*n* = 5/group): (1) untreated mice, (2) mice subjected to intraperitoneal administration (IP) of anti-CD73 antibody (50 μg/mouse) alone, (3) mice subjected to intraperitoneal administration (IP) of 5 × 10^6^ CAR-NK-92 cells alone, and (4) mice subjected to intraperitoneal administration (IP) of both 5 × 10^6^ CAR-NK-92 cells and 50 μg/mouse of anti-CD73 antibody. CAR-NK-92 cells were administered one time per week, 3 times in total. The anti-CD73 antibody was administered 24 h before each injection of CAR-NK-92 cells. Meanwhile, the mice that received NK cell therapy (groups 3 and 4) also received 2000 U rhIL-2 by IP via a single injection every two days. The length (L), width (W), and height (H) of the tumor were measured using a digital caliper, and the volume of the tumor was calculated using the formula: V = 0.52 × L× W × H. Body weights of the mice were also recorded during the treatment. At the end of the experiment, the mice were sacrificed and tumors were harvested for histologic analyses.

### Immunohistochemistry (IHC) studies

The harvested tumors were fixed in 10% neutral-buffered formalin, embedded in paraffin, and cut into 3–5 μm sections. NK-92 cells in tumors were detected by immunohistochemical staining using a mouse anti-human CD56 antibody (clone 56C04, ThermoFisher Scientific) at a 1:200 dilution. For the quantification of NK-92 cells in the tumors, the stained cells were counted in 5 randomly selected intratumoral fields of each slide under ×200 magnification.

### Statistical analysis

Data were presented as mean ± SEM. Statistical analysis was performed using Excel 2007 software (Microsoft office 2007). The difference between two groups was analyzed by a one-way ANOVA analysis. *P* < 0.05 was considered to be statistically significant.

## Additional file

Additional file 1: **Figure S1.** (**A**) Scheme of the synthesis of PBAE 447. (**B**) 1 H NMR spectra of PBAE 447 polymer in DMSO-d6. **Figure S2.** Cell viability of NK-92 cells after incubation with PBAE 447 polymer at various concentrations for 52 h. **Figure S3.** Flow cytometric analyses of NKG2D expression in NK-92 cells. NK-92 cells were stained with FITC-NKG2D and positive staining was analyzed. **Figure S4.** The mean fluorescence intensity (MFI) of NKG2D expression following 5 days’ continuous culture of NKG2D.CAR-NK-92 cells. **Figure S5.** (**A**) Cell viability of NK-92 cells after incubation with TGF-β1 for 24 h. (**B**) The effect of TGF-β1 treatment on NKG2D expression by NK-92 cells. **Figure S6.** (**A**) Percentage of NK cells producing IFN-γ intracellularly measured by flow cytometry. (**B**) Degree of degranulation of NK cells expressed as % CD107a^+^ cells. **Figure S7.** Cell viability of NK-92 cells after incubation with adenosine (ADO) at various concentrations for 24 h. **Figure S8.** Lytic activity of NK-92 cells against (**A**) GBM43, (**B**) GBM10, (**C**) A549 or (**D**) PC3 cells, in the presence of anti-CD73 antibody (10 μg/mL) and adenosine deaminase inhibitor (ADAi) EHNA (30 μM), respectively. **Figure S9.** CD73 expression on (**A**) A549 and (**B**) GBM10 cells after treatment with TGF-β1 for 24 h. **Figure S10.** (**A**) CD73 expression on K562 cells. (**B**) Lytic activity of NK-92 and piggyBac-NKG2D.CAR-NK-92 cells against CD73^-^ K562 cells. (DOCX 914 kb)

## Abbreviations

ADA: Adenosine deaminase
ADCC: Antibody-dependent cellular cytotoxicity
ADO: Adenosine
ADP: Adenosine diphosphate
AMP: Adenosine monophosphate
AMP^r^: Ampicillin resistance
ATP: Adenosine triphosphate
BGH PA: Bovine growth hormone polyadenylation signal
CAG: Chicken β actin
CAR: Chimeric antigen receptor
CMV: Human cytomegalovirus
ECM: Exracellular matrix
EF1A: Eukaryotic translation elongation factor 1 alpha 1
EHNA: Erythro-9-(2-hydroxy-3-nonyl)adenine
ENTPDase1: Ectonucleoside triphosphate diphosphohydrolase-1
IFN-γ: Interferon-γ
MDSC: Myeloid-derived suppressor cell
MFI: Mean fluorescence intensity
MHC: Major histocompatibility complex
MMP: Matrix metalloproteinase
NK: Natural killer
NKG2D: Natural killer group 2 D
NSG: Non-obese diabetic scid gamma
ORI: Origin of replication
PBAE: Poly(beta-amino ester)
rBG pA: Rabbit β-globin polyadenylation signal
TMZ: Temozolomide
T_reg_: Regulatory T cell
