# Supplementary material for: Purinergic targeting enhances immunotherapy of CD73+ solid tumors with piggyBac-engineered chimeric antigen receptor natural killer cells
Source: J Immunother Cancer. 2018 Dec 4;6:136. doi: 10.1186/s40425-018-0441-8 (PMC6278070; doi:10.1186/s40425-018-0441-8)
Supplement: Supplementary file 1 — Figure S1. (A) Scheme of the synthesis of PBAE 447. (B) 1 H NMR spectra of PBAE 447 polymer in DMSO-d6. Figure S2. Cell viability of NK-92 cells after incubation with PBAE 447 polymer at various concentrations for 52 h. Figure S3. Flow cytometric analyses of NKG2D expression in NK-92 cells. NK-92 cells were stained with FITC-NKG2D and positive staining was analyzed. Figure S4. The mean fluorescence intensity (MFI) of NKG2D expression following 5 days’ continuous culture of NKG2D.CAR-NK-92 cells. Figure S5. (A) Cell viability of NK-92 cells after incubation with TGF-β1 for 24 h. (B) The effect of TGF-β1 treatment on NKG2D expression by NK-92 cells. Figure S6. (A) Percentage of NK cells producing IFN-γ intracellularly measured by flow cytometry. (B) Degree of degranulation of NK cells expressed as % CD107a+ cells. Figure S7. Cell viability of NK-92 cells after incubation with adenosine (ADO) at various concentrations for 24 h. Figure S8. Lytic activity of NK-92 cells against (A) GBM43, (B) GBM10, (C) A549 or (D) PC3 cells, in the presence of anti-CD73 antibody (10 μg/mL) and adenosine deaminase inhibitor (ADAi) EHNA (30 μM), respectively. Figure S9. CD73 expression on (A) A549 and (B) GBM10 cells after treatment with TGF-β1 for 24 h. Figure S10. (A) CD73 expression on K562 cells. (B) Lytic activity of NK-92 and piggyBac-NKG2D.CAR-NK-92 cells against CD73- K562 cells. (DOCX 914 kb) [file 40425_2018_441_MOESM1_ESM.docx]

**Supplementary information**

**Purinergic targeting enhances immunotherapy of CD73^+^ solid tumors with piggyBac-engineered chimeric antigen receptor natural killer cells**

Jiao Wang^1^, Kyle B. Lupo^1^, Andrea M. Chambers^1^, Sandro Matosevic^1,2^

^1^Department of Industrial and Physical Pharmacy, Purdue University, West Lafayette, Indiana, United States

^2^ Center for Cancer Research, Purdue University, West Lafayette, Indiana, United States

**Running title:** CD73 targeting enhances immunotherapy with CAR-NK cells

**Keywords:** NK cells, adenosine, CD73, chimeric antigen receptor, cancer immunotherapy

**Corresponding author:** Sandro Matosevic, Purdue University, 575 Stadium Mall Drive, Robert E. Heine Pharmacy Building, West Lafayette, IN, 47907 USA. Phone: +1 765 494 1400. E-mail: sandro@purdue.edu


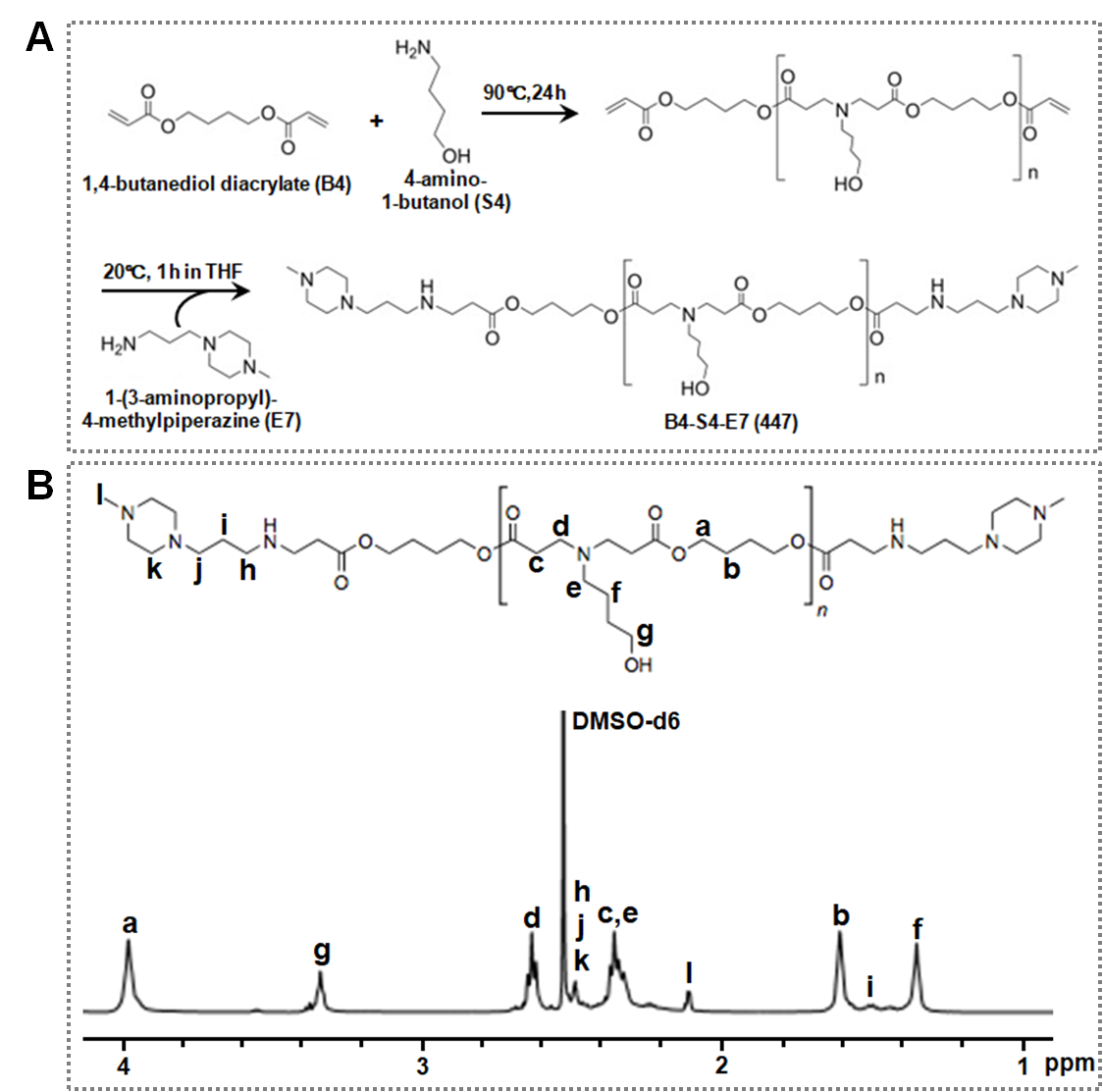


**Figure S1.** (**A**) Scheme of the synthesis of PBAE 447. (**B**) 1 H NMR spectra of PBAE 447 polymer in DMSO-d6.

1 H NMR spectra of PBAE447 was measured by NMR spectrometers ([Bruker DRX500-2](http://www.pinmrf.purdue.edu/instruments/drx500-2.shtml))


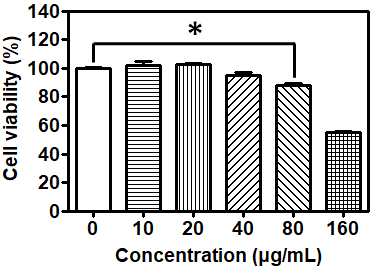


**Figure S2.** Cell viability of NK-92 cells after incubation with PBAE 447 polymer at various concentrations for 52 h. Data are presented as the mean ± SEM (n = 4). **P* < 0.05.

*In vitro* cytotoxicity was examined by the CCK-8 assay. Briefly, NK-92 cells were seeded into a 96-well plate (4 × 10^4^/well, in 100 μL complete cell culture medium), and incubated with PBAE at different concentrations (10, 20, 40, 80, and 160 μg/mL) for 52 h. Then, CCK-8 solutions were added (10 μL/well) into each well and the cells were incubated at 37 ℃ for another 1 h. After that, the absorbance was determined at 450 nm by a microplate reader (BioTek Synergy 4).


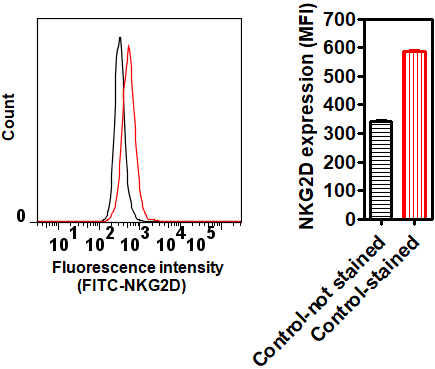


**Figure S3.** Flow cytometric analyses of NKG2D expression in NK-92 cells. NK-92 cells were stained with FITC-NKG2D and positive staining was analyzed. Data are presented as the mean ± SEM (n = 4).


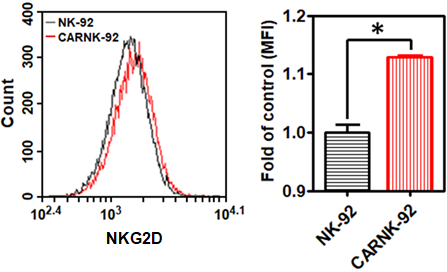


**Figure S4.** The mean fluorescence intensity (MFI) of NKG2D expression following 5 days’ continuous culture of NKG2D.CAR-NK-92 cells. Data are presented as the mean ± SEM (n = 3). **P* < 0.05.


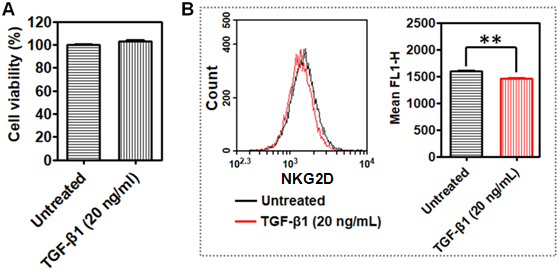


**Figure S5.** (**A**) Cell viability of NK-92 cells after incubation with TGF-β1 for 24 h. (**B**) The effect of TGF-β1 treatment on NKG2D expression by NK-92 cells. Data are presented as the mean ± SEM (n = 3). ** *P* < 0.01.

NK-92 cells were seeded into a 24-well plate (2 × 10^5^/well, in 500 μL complete cell culture medium), and incubated with TGF-β1 at a final concentration of 20 ng/mL for 24 h.

1. For cytotoxicity determination, 100 μL of cell culture medium was collected from each well and transferred into a 96-well plate. CCK-8 solutions were added (10 μL/well) into each well and the cells were incubated at 37 ℃ for another 1 h. After that, the absorbance was determined at 450 nm by a microplate reader (BioTek Synergy 4).
2. To investigate NKG2D expression, the remaining cells were collected and stained with FITC-conjugated NKG2D antibody (clone 1D11, BioLegend) and analyzed using a BD Accuri C6 Plus cytometer (Becton Dickinson).


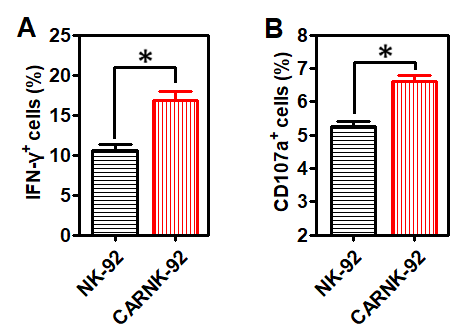


**Figure S6.** (**A**) Percentage of NK cells producing IFN-γ intracellularly measured by flow cytometry. (**B**) Degree of degranulation of NK cells expressed as % CD107a^+^ cells.


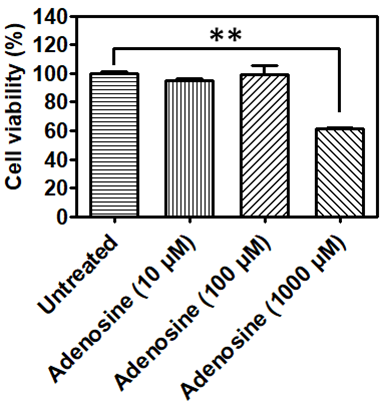


**Figure S7.** Cell viability of NK-92 cells after incubation with adenosine (ADO) at various concentrations for 24 h. Data are presented as the mean ± SEM (n = 4). ** *P* < 0.01.

*In vitro* cytotoxicity was examined by the CCK-8 assay. Briefly, NK-92 cells were seeded into a 24-well plate (2 × 10^5^/well, in 500 μL complete cell culture medium), and incubated with PBAE at different concentrations (10, 100, and 1000 μg/mL) for 24 h. Then, 100 μL of cell culture medium was collected from each well and put into a 96-well plate. CCK-8 solutions were added (10 μL/well) into each well and the cells were incubated at 37 ℃ for another 1 h. After that, the absorbance was determined at 450 nm by a microplate reader (BioTek Synergy 4).


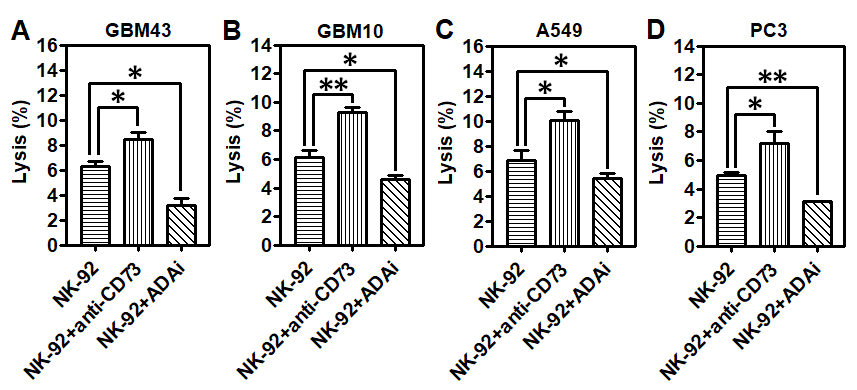


**Figure S8.** Lytic activity of NK-92 cells against (**A**) GBM43, (**B**) GBM10, (**C**) A549 or (**D**) PC3 cells, in the presence of anti-CD73 antibody (10 μg/mL) and adenosine deaminase inhibitor (ADAi) EHNA (30 µM), respectively. Data are presented as the mean ± SEM (n = 4). **P* < 0.05, ***P* < 0.01.

The lytic activity of NK-92 cells against GBM43, GBM10, A549 or PC3 cells in the presence of anti-CD73 (clone 7G2, ThermoFisher Scientific) or an adenosine deaminase inhibitor (EHNA hydrochloride) was analyzed using the 7-AAD/CFSE assay according to the procedure described earlier in Materials and Methods (2.9). The final concentrations of anti-CD73 antibody and EHNA hydrochloride were 10 μg/mL and 30 μM, respectively.


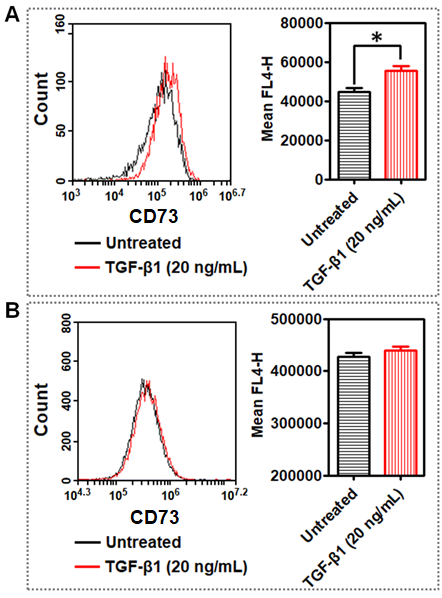


**Figure S9.** CD73 expression on (**A**) A549 and (**B**) GBM10 cells after treatment with TGF-β1 for 24 h. Data are presented as the mean ± SEM (n = 3). * *P* < 0.05.

A549 or GBM10 cells (2 × 10^5^/well, in 500 μL complete cell culture medium) were seeded into a 24-well plate overnight for attachment. Then, TGF-β1 was added into wells at a final concentration of 20 ng/mL. After incubation for 24 h, the cells were collected and stained with APC-conjugated mouse anti-human CD73 (clone AD2, BD Biosciences) and APC mouse IgG1, κ isotype (clone MOPC-21, BioLegend) for 30 min at 4 °C, respectively. Then, cells were assessed for expression of CD73 using a BD Accuri C6 Plus cytometer (Becton Dickinson).


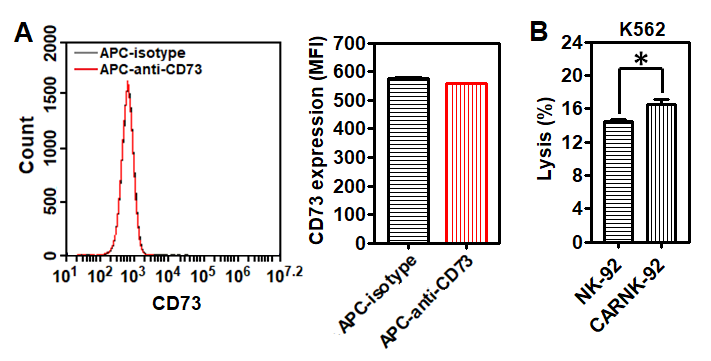


**Figure S10.** (**A**) CD73 expression on K562 cells. (**B**) Lytic activity of NK-92 and piggyBac-NKG2D.CAR-NK-92 cells against CD73^-^ K562 cells. Data are presented as the mean ± SEM (n = 4). **P* < 0.05.

Human chronic myeloid leukemia cells (K562) cells were purchased from ATCC and maintained in RPMI 1640 supplemented with 10% FBS, 100 U/mL penicillin, 100 µg/mL streptomycin.

1. For CD73 expression measurement, K562 cells (106/sample) were stained with APC-conjugated mouse anti-human CD73 (clone AD2, BD Biosciences) and APC mouse IgG1, κ isotype (clone MOPC-21, BioLegend) for 30 min at 4 °C, respectively. Then, cells were assessed for expression of CD73 using a BD Accuri C6 Plus cytometer (Becton Dickinson).
2. NK cell-mediated cytotoxicity against K562 was analyzed using a 7-AAD/CFSE assay (Cayman Chemical). Briefly, target cells (K562) were labeled with CFSE and seeded into 24-well plates. Then, effector cells (NK-92 or CAR.NK-92) were added at a E:T ratios of 10:1, and co-cultured with target cells for 4 h. Finally, the cells were collected and stained with 7-AAD and cytotoxicity was measured by ﬂow cytometry using a BD Accuri C6 Plus flow cytometer (Becton Dickinson).
